# Diabetes Mellitus is Associated With Higher COVID-19 Mortality Rates in Sub-Saharan Africa: A Systematic Review and Meta-analysis

**DOI:** 10.7759/cureus.26877

**Published:** 2022-07-15

**Authors:** Ben Bepouka, Ossam Odio, Donat Mangala, Nadine Mayasi, Madone Mandina, Murielle Longokolo, Jean Robert Makulo, Marcel Mbula, Jean Marie Kayembe, Hippolyte Situakibanza

**Affiliations:** 1 Infectious Diseases, University of Kinshasa, Kinshasa, COD; 2 Nephrology, University of Kinshasa, Kinshasa, COD; 3 Pulmonology, University of Kinshasa, Kinshasa, COD

**Keywords:** africa, meta-analysis, mortality, diabetes mellitus, covid-19

## Abstract

The rate of COVID-19-related mortality among patients with diabetes mellitus in Sub-Saharan Africa (SSA) is unknown. The current study aimed to determine the mortality rate of COVID-19 among diabetes patients in SSA. We performed a systematic review of research articles until July 1, 2021. A literature review was conducted in accordance with the PRISMA guidelines to gather relevant data. A random effects model was used to calculate odds ratios and 95% confidence intervals (CIs). We used Egger's tests and Begg's funnel plot to examine publication bias. The mortality rate of 7778 COVID-19 patients was analyzed using data from seven studies. The I^2^ test was used to determine the heterogeneity between studies. The meta-analysis revealed that diabetes mellitus was linked to a 1.39-fold increase in the risk of death among COVID-19 inpatients (95% CI: 1.02-1.76). According to our findings, there was no significant heterogeneity between studies, and there was no publication bias. The present review describes an association between diabetes mellitus and the risk of COVID-19 mortality in SSA.

## Introduction and background

The World Health Organization (WHO) designated coronavirus disease 2019 (COVID-19) a public health emergency on January 30, 2020. As of September 23, 2021, COVID-19 has infected 229 415 774 individuals worldwide, resulting in 4 699 359 deaths, with 8 180 555 infections and 206 137 deaths in Africa, resulting in a case fatality rate of 2.5 percent [1]. Symptomatic individuals appear with mild symptoms in approximately 80% of cases present with mild symptoms, 15% present with moderate-to-severe symptoms, and 5% present with critical symptoms [2]. Diabetes mellitus (DM) is one of the most common chronic diseases in the world, causing severe acute and chronic consequences for an estimated 463 million individuals in 2019 [3]. Diabetes mellitus accounts for a considerable share of consultations, complications, and deaths in Africa's epidemiological transition, where infectious diseases are giving way to noncommunicable diseases [4].

Diabetes has clearly become a hidden pandemic on the continent and is expected to worsen in the future. According to International Diabetes Federation (IDF) predictions, the prevalence of diabetes is expected to rise by 156 percent in Africa, 16 percent in Europe, 35 percent in North America and the Caribbean, and 84 percent in Southeast Asia by 2045 [5]. Although it is unclear whether patients with diabetes mellitus are more vulnerable to COVID-19, multiple studies and meta-analyses from throughout the world have found a link between severe COVID-19 infection or COVID-19 death and diabetes mellitus [6-10]. A recent meta-analysis pooled studies on mortality risk factors associated with hypertension and COVID-19, but there is not yet a meta-analysis of African studies on the link between diabetes and the likelihood of COVID-19-related death [11].

Several observational studies on predictors of mortality in COVID-19 patients have been undertaken in Africa, with mixed results regarding the role of diabetes as a comorbidity in death. Diabetes has been demonstrated to be a predictor of mortality in some studies [12-14], although this idea has not been supported in others [15-18]. Varied study designs and populations provide different estimates and impact sizes in these published publications. Because of this unpredictability, a complete and systematic examination is needed. The purpose of this study was to conduct a systematic review and meta-analysis to investigate the link between diabetes mellitus and mortality among COVID-19 patients in Africa.

## Review

Methods

Data Sources

The Preferred Reporting Items for Systematic Reviews and Meta-Analyses (PRISMA) checklist was used to report this article. Between March 1st, 2020, and July 1st, 2021, we conducted a retrospective systematic review of PubMed, Google Scholar, and Web of Science databases. Full terms for "mortality," "COVID-19," "Diabetes mellitus," and "Sub-Saharan Africa" were included in the search terms. The reference lists of the included studies were manually searched to identify additional relevant studies and review papers. Table 1 contains a full description of our search strategy. The abstracts of selected articles were screened for relevance by two independent investigators. The full texts of the remaining studies were then assessed using the inclusion and exclusion criteria.

**Table 1:** The search strategy in PubMed COVID-19: Coronavirus disease 2019; SARS-CoV-2: Severe acute respiratory syndrome coronavirus 2 Adapted from [11]

Search concept	Search Terms
COVID-19	COVID-19 OR SARS-CoV-2
Diabetes mellitus	Hyperglycemia OR diabetes
mortality	Mortality OR lethality OR fatal outcome Death?
Sub-Saharan Africa*	“Africa South of the Sahara"* OR Central Africa* OR Western Africa* OR Eastern Africa* OR Southern Africa* OR Benin* OR*Tanzania* OR Togo* OR Uganda* OR Zimbabwe* OR Cameroon* OR Cape Verde* OR Congo * OR Democratic republic of the Congo* OR Cote d'Ivoire* OR Ghana* OR Lesotho* OR Mauritania* OR Nigeria* OR Atlantic Islands* OR Senegal* OR Sudan* OR South Sudan* OR Swaziland* OR Zambia* OR Angola* OR Botswana* OR Gabon* OR Mauritius* OR Namibia* OR Seychelles* OR South Africa* OR Equatorial Guinea*OR Benin* OR Burkina Faso* OR Burundi* OR Central African Republic* OR Chad* OR Comoros* OR “Democratic Republic of the Congo"* OR Eritrea* OR Ethiopia* OR Gambia* OR Guinea* OR Guinea-Bissau* OR Kenya* OR Liberia* OR Madagascar* OR Malawi* OR Mali* OR Mozambique* OR Niger* OR Rwanda* OR Sierra Leone* OR Somalia*
	All the above sets (1-4) were combined with “AND”

Study Selection

**Figure 1:**
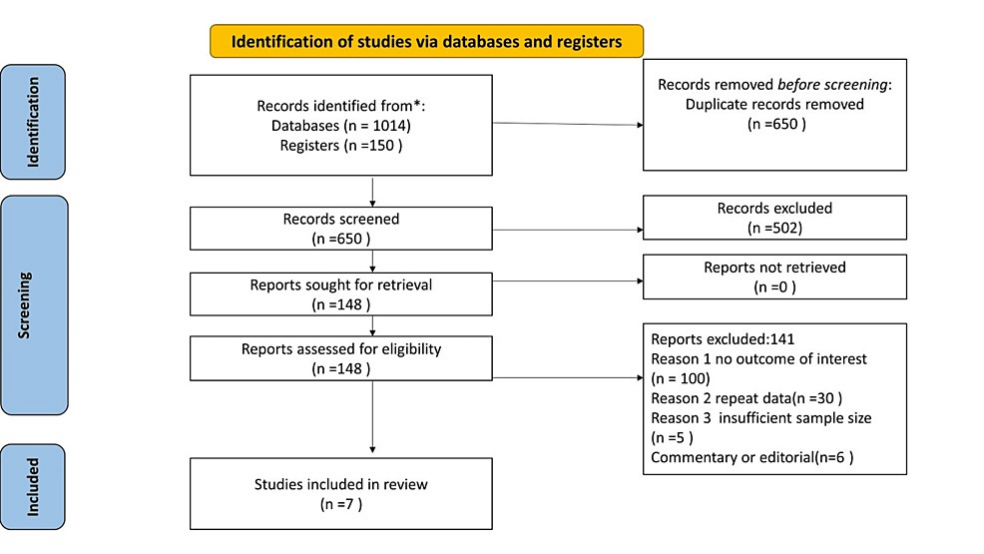
Prisma flow chart showing study selection

Based on the appropriateness of the title and abstract, two independent investigators chose potentially eligible studies. The full texts were then examined in accordance with the eligibility requirements (Figure 1). The analysis excluded commentaries, case reports, family studies, and pediatric studies. Clinical studies that did not clearly state death as a possible consequence were also excluded. Apart from the articles included in our meta-analysis, we also selected some existing meta-analyses and global studies on the association between diabetes mellitus and COVID-19 mortality risk to allow comparative analysis.

Data Extraction

Two researchers independently utilized our data extraction form, which included information on authors, year of publication, nation, study design, study location (number of study sites), sample size, age, sex, diabetic history, and result. For the existing meta-analyses, we included the information on pooled mortality or severity. Another researcher verified the accuracy of the completed data extraction forms and compared the data per article to eliminate duplicates and address inconsistencies.

Quality Assessment

The methodological Items for Non-Randomized Research (MINORS) list was used by two independent reviewers to evaluate the methodological merit of the included studies. Each of the twelve MINORS elements was given a score of 0 if it was not reported, 1 if it was reported insufficiently, and 2 if it was reported adequately, for a maximum score of 24 points. If the overall score was 17 or greater, the study was considered "high quality." If the total score was 17 or less, the study was considered "poor quality" [19,20].

Data Analysis

The link between diabetes mellitus and death in COVID-19 participants was evaluated using the odds ratios (ORs) with a 95% confidence interval (CI). To examine study heterogeneity, the I^2^ statistic and the X^2^ test were used. For the X^2^ test, a Cochran's Q p-value of 0.10 revealed considerable heterogeneity between trials. Low heterogeneity was defined as an I^2^ value less than 25%, moderate heterogeneity was defined as an I^2^ value between 25% and 50%, and high heterogeneity was defined as an I^2^ value greater than 50% [21]. Forest plots were utilized to depict the effect estimates from the research. We used Egger's test to determine whether there was any publication bias. A p-value < 0.05 indicated statistical significance. STATA software version 14 (StataCorp LP, College Station, TX) was used for statistical analysis.

Results

Study Selection and Characteristics

The flow chart shows the studies included in the present study (Figure 1 above). Based on the criteria described previously, a total of seven studies (all retrospective except one and all of the excellent quality) involving 7778 COVID-19 patients from six Sub-Saharan African (SSA) countries were ultimately included in our meta-analysis (Table 2). The research was performed and published until July 2021. The sample sizes of verified COVID-19 patients ranged from 105 to 2617. The prevalence of diabetes mellitus among the patients in the studies ranged from 6% to 49% [12-18]. The available meta-analysis on the association of diabetes mellitus and mortality risk with COVID-19 reported ORs ranging from 1.75 to 9.9. These meta-analyses pooled studies from around the world, mostly from China, without including African studies [6-10, 22-25]. In addition, an ecological investigation in Africa also reported that diabetic patients had a high risk of death in a population of 1 million Africans [26].

**Table 2:** Characteristics of included studies, other meta-analysis, and global studies OR: odds ratio; RR: risk ratio; NA: not applicable; -: not clearly reported; GHS: Global health security detection index (Weighted sum of all the GHS data normalized to a scale of 0 to 100, where 100 = best health security condition Matangila et al 2020 [15]; Bepouka et al. 2020 [17]; Nachega et al. 2020 [18]; Abraha et al. 2021 [12]; Jaspard et al. 2021 [16]; Osibogun et al. 2021 [13]; Ghada et al. 2021 [14]; Aggarwal et al. [22]; Huang et al. [7]; Miller et al. [23]; Kumar et al. [6]; Varikasuvu et al. [8]; Wu et al. [24]; Guo et al. [25]; Montovani et al. [10]; Bouba et al. [26]

Studies	Design of study	Countries	Size	Setting	Male (%)	Age [median, (interquartile range), mean (SD)]	Diabetes (%)	Outcome	Pooled analysis of outcome
Matangila et al. 2020	retrospective	DRC	160	Single hospital in Kinshasa	51.0	54(38-64)	19	mortality	NA
Bepouka et al. 2020	retrospective	DRC	141	Single hospital in Kinshasa	67.4	49.6+-16.5	17	mortality	NA
Nachega et al. 2020	retrospective	DRC	766	7 largest health facility in Kinshasa	65.6	46(34-58)	14	mortality	NA
Abraha et al. 2021	retrospective	Ethiopia	2617	Single hospital in Mekelle city	63.3	29(24-38)	3.1	mortality	NA
Jaspard et al. 2021	prospective	Guinea and Burkina	1805	3 hospitals in Burkina and Guinea	64.0	41(30-57)	12	mortality	NA
Osibogun et al. 2021	retrospective	Nigeria	2184	10 centres in Lagos	65.8	43(35-55)	6	mortality	NA
Ghada et al. 2021	retrospective	Sudan	105	Single health facility	62.9	-	49	mortality	NA
Aggarwal et al. 2020	Meta-analysis Of 16 studies	China	2564	NA	-	-	10.3	Poor outcome; Mortality	OR:2.03 (CI 95%:1.29-3.20); OR:2.60 (CI 95%:1.96-3.45)
Huang et al. 2020	Meta-analysis of 30 studies	Worldwide (More in China)	6452	NA	-	-	-	Poor outcome; Mortality	OR: 2.38 (CI 95 %:1.88-3.03) ; OR:2.12 (CI 95%: 1.44-3.11)
Miller et al. 2020	Meta-analysis of 16 studies	China	1832	NA	53	53	14.4	Mortality	OR:9.9(CI 95%: 6.1-14.5)
Kumar et al. 2020	Meta-analysis of 33 studies	Worldwide (more in USA, China and France)	16003	NA	54	52.6±17.4	11.2	Severe COVID; Mortality	OR: 2.75 (CI 95%: 2.09-3.62) ; OR:1.90 (CI 95%: 1.37-2.64)
Varikasuvu et al. 2021	Meta-analysis of 47 studies	Worldwide (more in China)	13268	NA	-	-	17.8	Mortality	OR: 2.32 (CI 95%: 1.90-2.83)
Wu et al. 2021	Meta-analysis of 9 studies	China	926	NA	-	-	-	Mortality	OR: 1.75 (CI 95%: 1.31-2.36)
Guo et al. 2020	Meta-analysis of 9 studies	China	1070	NA	48	44-61	-	Mortality	RR: 2.96(2.31-3.79)
Montovani et al. 2020	Meta-analysis of 83 studies	Worldwide( more in China, Italy, France, United Kingdom,Australia, United States	78874	NA	52.1	-	14.34	Severity; Mortality	OR: 2.10(1.71-2.57); OR:2.68(2.09-3.44)
Bouba et al. 2021	Ecological investigation	54 African countries	Study for 1 million population in Africa	NA	-	-	-	Mortality	Factors associated with covid-19 death per 1 million population in Africa are the prevalence of diabetic patients, the number of nurses per 1000 populations and the global health security (GHS) detection index

Analysis of Mortality

COVID-19 patients with diabetes mellitus had a higher risk of death (OR 1.39, 95% CI: 1.02-1.76) than those without diabetes mellitus.

Heterogeneity Across Studies

**Figure 2:**
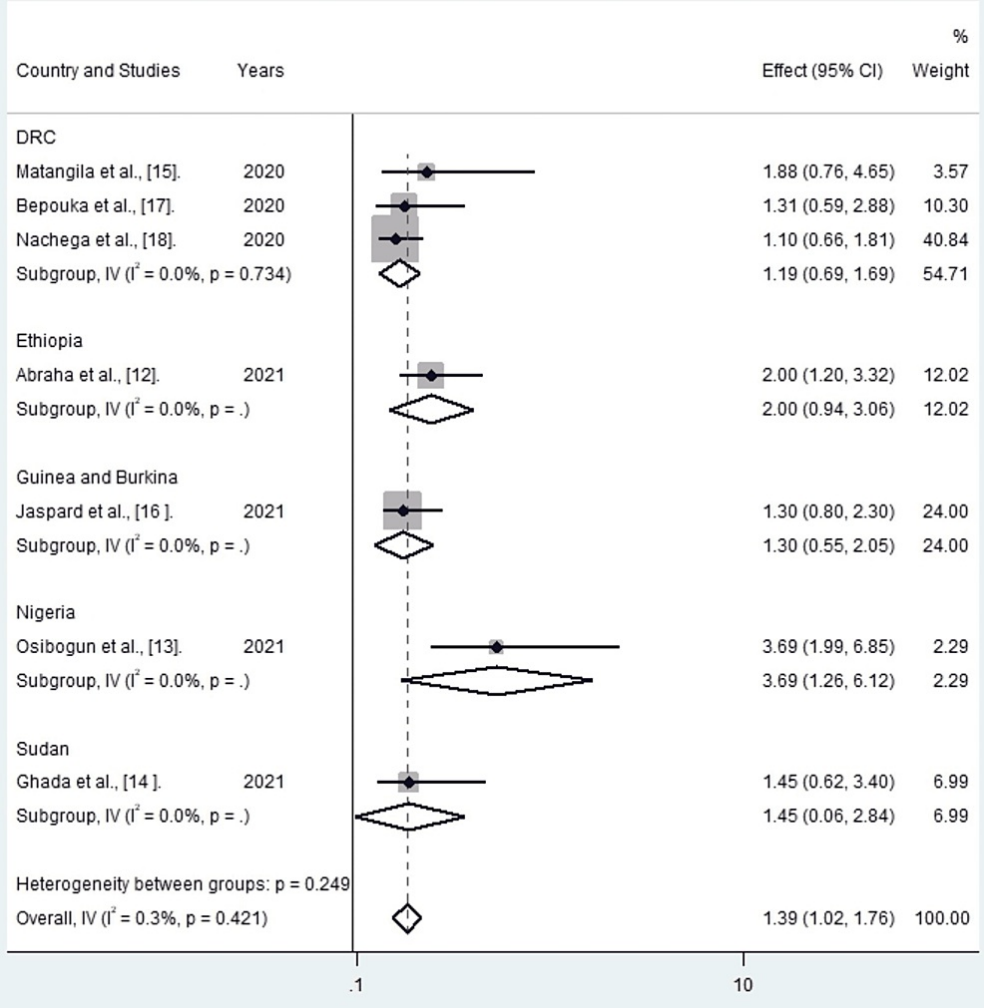
Meta-analysis results of the relationship between diabetes mellitus and risk of death due to COVID-19

There was no evidence of heterogeneity between studies (I^2^=0.3%, Cochrane Q test 6.02 p=0.421) (Figure 2).

Publication Bias

**Figure 3:**
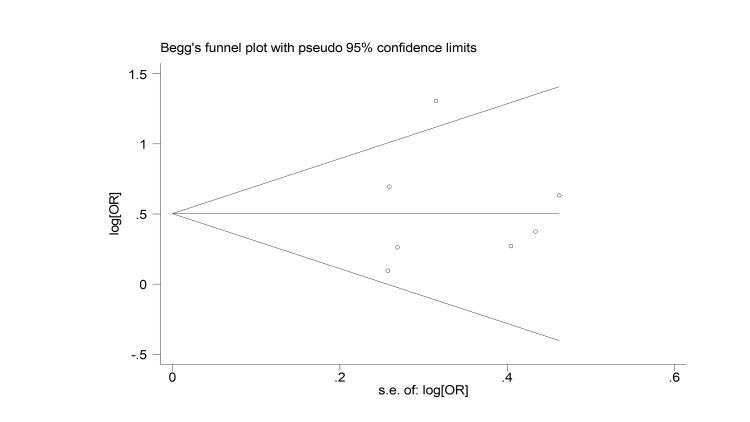
Begg’s funnel plot for the assessment of publication bias. The outcome of mortality.

A visual inspection of Begg’s funnel plot revealed symmetry, indicating a low risk of publication bias (Figure 3). Egger's test also revealed no publication bias (p=0.80).

Discussion

Our findings show that having diabetes can greatly aggravate COVID-19's clinical course. Overall, we found a higher risk of mortality in COVID-19 patients with diabetes than among those without diabetes in SSA. Other meta-analyses and ecological investigations also confirm this mortality risk [6-10, 22-26]. Although it is unknown whether people with diabetes are more susceptible to COVID-19, multiple investigations found a higher risk of severe COVID-19 in patients with diabetes [27-31]. Several hypotheses may explain this relationship. The pathophysiologic and virologic mechanisms explaining the substantial link between preexisting diabetes and an elevated risk of severe/critical illness or in-hospital mortality with COVID-19 are still unknown. Underlying metabolic changes, chronic inflammation, and/or attenuation of innate and adaptive immune responses (e.g., impaired leukocyte phagocytosis, impaired neutrophil chemotaxis, and bactericidal activity, and impaired cell-mediated innate immunity) are the causes of the increased severity of COVID-19 among patients with diabetes, predisposing them to infectious events of varying severity. One mechanism connecting diabetes mellitus to higher mortality rates among COVID-19 patients is the presence of an underlying pro-inflammatory milieu, as suggested by the finding that circulating levels of some cytokines, such as interleukin-6 (IL-6), are higher in COVID-19 patients with diabetes mellitus. Interleukin-1 and interleukin-6, two pro-inflammatory cytokines, have been linked to greater levels of inflammation in people with diabetes [32]. In COVID-19 individuals with diabetes, it was also discovered that many markers, such as C-reactive protein, fibrinogen, and D-dimer, were raised. Therefore, this condition may worsen the cytokine storms in COVID-19 and cause a more serious illness [33].

Another mechanism linking diabetes to a more severe outcome in COVID-19 patients is that patients with diabetes are frequently overweight or obese, which may also contribute to a worse prognosis in restrictive lung disease [34]. Another reason is that people with diabetes have a higher risk of respiratory infections because of the weakened immune system, especially innate immunity [35]. Even temporary hyperglycemia can impair innate immune responses to infection for a short time. Poorly controlled blood glucose and chronic hyperglycemia are associated with defects in lymphocyte proliferation as well as impaired monocyte/macrophage and neutrophil function [36]. In addition, hyperglycemia in pulmonary vessels at the time of infection has been shown to increase the local replication of influenza viruses, possibly SARS-CoV-2, in lung tissue. Furthermore, patients with diabetes may have higher levels of angiotensin-converting enzyme 2 (ACE-2) expression, which facilitates viral absorption and increases the risk of serious infection [37].

Limitations

These meta-analysis findings should be viewed with caution. First, we were constrained in our capacity to draw causal inferences due to the observational and retrospective nature of the selected studies. As a result, the findings could be influenced by reverse causality bias. Secondly, the authors included age, diabetes, sex, and mortality in the parameters for the systematic review. There could be other parameters that would influence the mortality in this study. Thirdly, most of the studies in our meta-analysis were conducted in hospitals or tertiary care facilities and our findings might not be applicable to patients outside of these settings. Despite these flaws, our research has several significant advantages. To guarantee that all relevant and published research was found, we conducted thorough database searches. There was no evidence of heterogeneity among the included studies.

## Conclusions

In patients with diabetes in Sub-Saharan Africa, the authors found an elevated risk of COVID-19 death. Diabetes mellitus is one of the conditions that should be actively monitored in the triage of high-risk COVID-19 patients. Patients with diabetes should be subjected to additional prevention and vaccination activities to help reduce in-hospital mortality.
